# Cross-Silo, Privacy-Preserving, and Lightweight Federated Multimodal System for the Identification of Major Depressive Disorder Using Audio and Electroencephalogram

**DOI:** 10.3390/diagnostics14010043

**Published:** 2023-12-25

**Authors:** Chetna Gupta, Vikas Khullar, Nitin Goyal, Kirti Saini, Ritu Baniwal, Sushil Kumar, Rashi Rastogi

**Affiliations:** 1 Chitkara University Institute of Engineering and Technology, Chitkara University, Rajpura 140417, Punjab, India; chetna1003cse.phd21@chitkara.edu.in (C.G.); vikas.khullar@gmail.com (V.K.); 2 Department of Computer Science and Engineering, School of Engineering and Technology, Central University of Haryana, Mahendergarh 123031, Haryana, India; sushil.kumar@cuh.ac.in; 3 Department of Electronics and Communication Engineering, University Institute of Engineering and Technology, Kurukshetra University, Kurukshetra 136119, Haryana, India; kirtisainiuiet@kuk.ac.in; 4 Department of Computer Science, Jyotiba Phule Government College, Radaur, Yamunanagar 135133, Haryana, India; baniwalritu@gmail.com; 5 Department of Computer Applications, Sir Chottu Ram Institute of Engineering & Technology, Ch. Charan Singh University, Meerut 250001, Uttar Pradesh, India; rastogi.rashi4@gmail.com

**Keywords:** major depressive disorder, federated learning, deep learning, Bi-LSTM, IIDs, non-IIDs, electroencephalography, audio

## Abstract

In this day and age, depression is still one of the biggest problems in the world. If left untreated, it can lead to suicidal thoughts and attempts. There is a need for proper diagnoses of Major Depressive Disorder (MDD) and evaluation of the early stages to stop the side effects. Early detection is critical to identify a variety of serious conditions. In order to provide safe and effective protection to MDD patients, it is crucial to automate diagnoses and make decision-making tools widely available. Although there are various classification systems for the diagnosis of MDD, no reliable, secure method that meets these requirements has been established to date. In this paper, a federated deep learning-based multimodal system for MDD classification using electroencephalography (EEG) and audio datasets is presented while meeting data privacy requirements. The performance of the federated learning (FL) model was tested on independent and identically distributed (IID) and non-IID data. The study began by extracting features from several pre-trained models and ultimately decided to use bidirectional short-term memory (Bi-LSTM) as the base model, as it had the highest validation accuracy of 91% compared to a convolutional neural network and LSTM with 85% and 89% validation accuracy on audio data, respectively. The Bi-LSTM model also achieved a validation accuracy of 98.9% for EEG data. The FL method was then used to perform experiments on IID and non-IID datasets. The FL-based multimodal model achieved an exceptional training and validation accuracy of 99.9% when trained and evaluated on both IID and non-IIID datasets. These results show that the FL multimodal system performs almost as well as the Bi-LSTM multimodal system and emphasize its suitability for processing IID and non-IIID data. Several clients were found to perform better than conventional pre-trained models in a multimodal framework for federated learning using EEG and audio datasets. The proposed framework stands out from other classification techniques for MDD due to its special features, such as multimodality and data privacy for edge machines with limited resources. Due to these additional features, the framework concept is the most suitable alternative approach for the early classification of MDD patients.

## 1. Introduction

Mental disorders are a major global health problem as they contribute significantly to the global burden of disease and have a major impact on people’s social and economic well-being. According to the World Health Organization (WHO), an estimated 264 million women and men of all ages suffer from a mental disorder, which means that up to 27% of the world’s population is affected by mental health problems at some point in their lives [1]. According to current WHO reports from 2023, an estimated 5% of adults worldwide suffer from depression [2]. Major depressive disorder (MDD) is a mental illness that has a significant impact on general behavior, emotions, and cognitive abilities [3]. In addition, it has been difficult to transfer diagnostic descriptions from clinically established nosology to various biological indices. We need new methods to accurately diagnose depression, including biomarkers [4,5,6,7,8]. According to the literature, depressed people release fewer neurotransmitters and have lower concentrations of synaptic receptors compared to healthy people [9,10].

Electroencephalography (EEG) is the conventional method used to detect brain activities which helps us in further diagnosis of brain diseases [11]. On the other hand, audio recordings are an unconventional method that can be used to recognize the frequency of valence and arousal in a person’s voice. Machine learning (ML) and deep learning (DL) are used to create a centralized model for different fields such as healthcare, agriculture, smart industry, intelligent management, and traffic and environmental monitoring systems [12]. Centralized models perform better because they have access to more data. Merging model parameters and training on the device only increases training time in federated learning (FL) in terms of model communication and aggregation [13,14,15]. For platforms focused on real-time and continuous monitoring of mental health problems, including depression, these trade-offs may be significant. Despite the fact that FL [16,17] has been used for a variety of healthcare services, there is a dearth of research that has utilized FL for privacy-preserving multimodal analysis to assess MDD.

Lightweight FL describes the establishment of technologies and approaches that reduce the amount of computational and communication resources required to build MDD models on mobile devices or distributed platforms [18]. It aims to make federated learning efficient and flexible in contexts with minimal resources [19]. It could be used in many different domains that require fast analysis of large amounts of distributed data [20]. In the field of FL, there is still a lack of studies on MDD classification. These challenges need to be addressed for FL to be used successfully in the real world for people with MDD. FL could, therefore, provide a private and secure system for a sensitive disease such as MDD, as many hospitals do not share their data for privacy reasons [21].

Major depressive disorder is a severe form of depression. However, there are many conventional methods for identifying MDD that are far less than optimal. AI-trained models have helped to develop more accurate and robust models for detecting MDD. Due to the sensitivity of medical data, it is difficult to share all patient information on a centralized device. Therefore, in this work, we have developed a privacy-preserving federated learning-based multimodal [22] system after studying the literature and research on MDD detection. Thus, in this work, a privacy-preserving multimodal FL system for MDD classification is developed using audio and EEG data. By having a local model at each node, the efficiency of a federated learning-based node is not significantly affected when connectivity is interrupted. It also reduces the communication overhead, which is the main drawback of algorithms based on deep learning or machine learning.

Researchers have developed a range of neural network-based machine learning and deep learning models to differentiate between MDD and non-MDD individuals. With developments in artificial intelligence, these models may be able to identify MDD subjects reliably and with high accuracy. Cai et al. [10] used four machine learning methods, Support Vector Machine (SVM), K-Nearest Neighbour (KNN), Classification Tree (CT), and Artificial Neural Network (ANN), to classify depression based on EEG signals and achieved a significant accuracy of 79.27% with the KNN algorithm. Orabi et al. [6] processed many deep learning algorithms to access the results of “X, formerly Twitter” data for depression detection and found that the Convolutional Neural Network (CNN) based model performed well compared to the Recurrent Neural Network (RNN) based model. Several techniques for recognizing various mental disorders are described in Table 1. However, finding a balance between data accuracy and privacy is a major problem. While the technology may detect diseases with great accuracy, there may be privacy issues.

Lam et al. [5] tested a transformer model and a 1D-CNN model for identifying depressed and non-depressed individuals using audio and text data, which performed well compared to other state-of-the-art depression detection systems. Zogan et al. [24] proposed a hybrid deep learning model using Tweeter data for detecting depressed individuals and achieved a good prediction with an F1 score of 89%. Mousavian et al. [32] developed depression detection based on resting-state MRI and structured MRI images using a machine learning model.

With the development of social media, people started sharing their emotions and feelings by posting their messages on different platforms like Facebook, “X, formerly Twitter”, WhatsApp, etc. Therefore, to diagnose and control depression, several optimized machine and deep learning algorithms were developed using social media data [6,24,29]. Many resources restrict access to other users, so data must be shared in a centralized location for privacy and sensitivity. The proposed study on the establishment of DL algorithms in a federated learning setting to improve data privacy has been emphasized in Table 1, Table 2 and Table 3.

The research gap identified in Table 1, Table 2 and Table 3 shows that considerable accuracy has been achieved in previous work but at the cost of privacy and resource utilization. Multimodal frameworks are expected to provide more precise and accurate results as the unique characteristics of each trained DL framework are considered across different MDD datasets [42,43,44]. Therefore, in the proposed framework, a multimodal system is developed using audio and EEG data. A major limitation of DL algorithms is the confidentiality of patient mental health data. Therefore, decentralized training with federated learning helps to protect the privacy of the data. The main limitation in centralized training of DL algorithms is the high communication cost required to transfer MDD-related data between the client and the server. The datasets in centralized DL are not IID. For non-ID datasets, independence means that all sample values are independent and not identically distributed. FL works with both IID and non-ID datasets. The aims of this research were as follows:

1. To develop and analyze baseline EEG and audio-based multimodal systems for the classification of MDD.
2. To implement and analyze privacy-preserved FL multimodal systems for the classification of MDD using EEG and audio databases.
3. To analyze the impact of identical and non-identical multimodal Cross-Silo databases on the FL-based MDD classification system.

The following sections of this work are structured as follows. Section 2 describes the associated work done in the suggested study using comparative analysis tables and research gap data. Section 3 mathematically defines the materials and methods used in the proposed study, as well as their subsections which include the proposed FL framework, DL methods, data collection details, multimodal framework, and methodology used. The outcomes of the base DL models and the suggested FL multimodal employing EEG and audio data are presented extensively in Section 4. Finally, Section 5 represents the proposed study’s conclusion.

## 2. Materials and Methods

**Figure 1:**
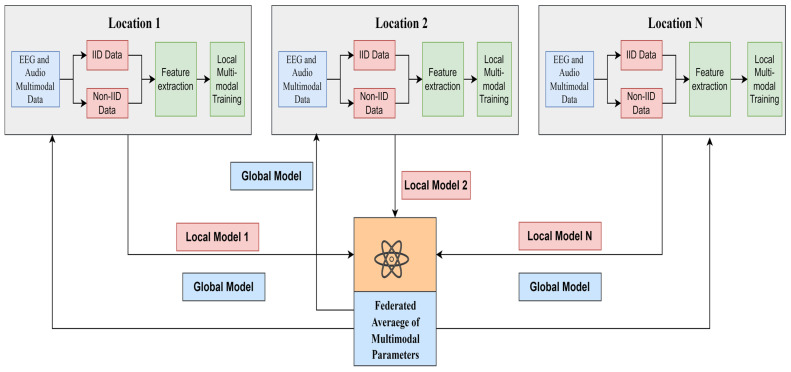
Proposed methodology workflow.

The following section provides examples of the procedures and methods used in the proposed work. In this study, a federated deep learning framework is presented to solve privacy issues related to depression classification. The framework uses a decentralized client-server architecture to ensure the confidentiality of all identically and non-identically distributed (IID and non-IID) client data. This research was conducted to classify MDD patients and healthy control subjects. For this purpose, we first collected an online dataset containing MDD data from EEG and audio recordings of many subjects. Subsequently, these data were preprocessed for further training and finally analyzed using our proposed framework. The results are presented below in Figure 1.

The privacy-preserving Multimodal Federated Learning (MFL) model was developed to achieve a robust model with the security of raw data by building a model on local devices. The audio and EEG data from MDD and healthy control subjects were collected in an online dataset. Subsequently, the data were pre-processed and divided into IID and non-IID datasets, which were then forwarded to the implementation phase. First, the DL algorithms Bidirectional Long-Term Memory (Bi-LSTM), Long Short-Term Memory (LSTM), and Convolutional Neural Network (CNN) were applied separately to audio and EEG datasets. In addition, the DL algorithm Bi-LSTM was implemented to create a multimodal system from audio and EEG datasets. Finally, the multimodal FL framework was developed to create a privacy-sensitive system for MDD subjects.

### 2.1. Proposed Multimodal Federated Learning Framework for MDD Classification

In general, collaborative learning can be applied to the simultaneous processing of large amounts of data on independently generated datasets with data processing nodes that have approximately similar computational power. Data sharing may also require collaborative learning with significant communication capacity and high data security. Federated learning is a collaborative machine learning architecture that enables data evaluation, such as neural network (NN) training, directly on the data storage device. Only the result parameters, such as changed weights of the NN, are imported into the neural network to create an aggregated analysis framework in FL. Therefore, compared to regular distributed systems, FL systems do not collect data in a centralized data warehouse.

**Figure 2:**
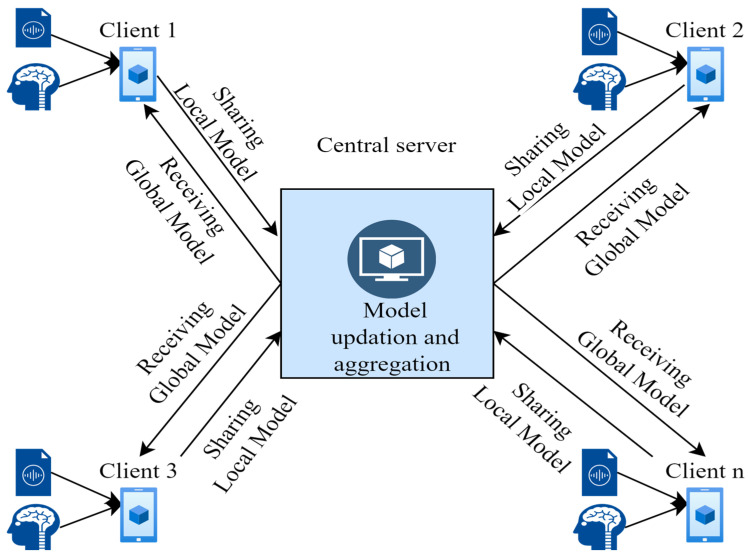
Multimodal Federated Learning based MDD detection using EEG and audio data.

The proposed FL framework for MDD classification is shown in Figure 2. For federated machine learning or deep learning, the proposed framework involves a client-server architecture, and the given tasks need to be performed on the client and server sides with separate programming codes. N clients were connected to a server in a federated cluster, each with an individual set of programming languages. In the current study, the proposed federated learning framework for MDD identification, including a client-server architecture, was implemented using Python programming libraries led by Keras and TensorFlow.

The server, the communication setting, and the clients are the three essential elements of a FL architecture. The formal FL explanation includes N clients (K1, K2, … KN) who have their datasets (D1, D2, … DN), so the full dataset is represented as DN = D1 ∪ D2 ∪…∪ DN. All shared datasets DN’s are pooled together in a standard decentralized training method to develop a deep learning framework. In the FL, each client’s data Di is trained independently, and each trained model collaborates without disclosing any client information due to other clients Ki or the server S. Further, the FL averaging structure is discussed below in Algorithm 1.
**Algorithm 1.** Federated AveragingK—number of clients from 1 to n B—minimum batch size E—number of epochs F—fractions of clients **Server function—FedAvg** Initialize global weights w
for round i = 1, 2…do  M ← max (F.K, 1)  $V_{t}$ ← (random sets of M clients)  for client k € $V_{t}$ do parallel  ClientUpdate(k, $w_{t}$)   $w_{t+1}\leftarrow {\Sigma^{K_{{k=1}^{Wk}}}}_{t+1}$
  end end **Client function—ClientUpdate(k,**
w**)** B ← Split data into batches For each local epoch i from1 to E do   Update client w
Return w to server

### 2.2. Deep Learning Methods

The DL techniques can help in the diagnosis of mental health issues using data from social media, clinical health records, and mobile device sensors. The centralized DL methods are widely used to identify and classify depression, and many researchers have developed robust models. Due to their ability to easily work on big data problems, in this work, we have used LSTM, CNN, and Bi-LSTM for preparing our base models.

In the CNN algorithm, a convolutional layer loops across the width and length in two dimensions. When the data inputs are Mel Frequency Cepstral Coefficients, whose dimensions represent duration and bandwidth, respectively, this does not apply. By addressing Mel Frequency Cepstral Coefficients (MFCC) in the depression detection problem, one-dimensional convolution is more appropriate than two-dimensional convolution. Convolution across the frequency axis is applied in the suggested model. As a result, the model is able to provide features that represent a brief temporal association. The configuration of a 1D CNN model requires forward and backward propagation layers; this is referred to as Multi-Layer Perceptron (MLP). In CNN layers, the forward propagation layer is presented as follows: $X_{k}^{l}=b_{k}^{l}+\sum_{i=1}^{n_{l-1}}\;conv1D(w_{ik}^{l-1},s_{i}^{l-1})$ where $X_{k}^{l}$ is the input, $b_{k}^{l}$ is the bias factor at $k^{th}$ the neuron of layer l, $w_{ik}^{l-1}$ is the kernel, and $s_{i}^{l-1}$ is the output of $i^{th}$ neuron. So, the output can be expressed as $Y_{k}^{l}=f(X_{k}^{l})$.

Long Short-Term Memory (LSTM) is specially used for sequential data like time series, waves, speech, text, and so on. LSTM is designed to overcome the long-term dependencies of Recurrent Neural Networks and provides more accurate predictions by remembering the information for a long time. It includes three gates; input gate, output gate, and forget gate. All of these gates generate values as 0 or 1, where 0 denotes that the gate is blocked, and no information is passing and 1 denotes the opposite. In LSTM, the representation of the Forgot Gate, *R*, is $R_{t}=\sigma \left(W_{f}.\left[C_{t-1,}h_{t-1},a_{t}\right]b_{f}\right)$. Input gate, *P*, is defined as $P_{t}=\sigma \left(W_{i}.\left[C_{t-1,}h_{t-1},a_{t}\right]b_{i}\right)$, and Output gate, *T*, is described as $T_{t}=\sigma \left(W_{o}.\left[C_{t-1,}h_{t-1},a_{t}\right]b_{o}\right)$.

Bidirectional LSTM is referred to as an updated version of LSTM because it works for information transmission in both directions, forward and backward at the same time. In this way, it helps to better predict the output value. The model can learn from both the present and the future time steps thanks to BiLSTM. We used BiLSTM to demonstrate the depression identification and detection system because we wanted to extract the properties of EEG and sound data. Therefore, it calculates the output value jointly as $D_{f}=\sigma \left(W_{f}\left[x_{n},h_{n-1}^{1},h_{n-1}^{2}\right]+b_{f}\right)$.

### 2.3. Multimodal Architecture

A data representation that incorporates information from various sources is known as a multimodal representation. Multimodality refers to obtaining most of the details of data by using multiple techniques, so that the deep learning algorithm may be trained effectively to produce superior outputs [45]. As each modality operates differently, merging them is difficult, and the beginning of each modality requires a different pattern. Stacking numerous diverse datasets may result in improved performance compared to individual models. A growing amount of multimodal data is being transferred through internet connections as an outcome of the expansion of the network and a wide range of smart gadgets in the past few years. Therefore, a larger variety of multimodal scenarios for applications are arising [46].

The recent achievement of large frameworks and their multimodal variations, in particular, highlights AI’s potential in multimodal core systems. The joint representations are methods that combine unimodal representations into a multimodal environment. Joint representations are employed in tasks that contain multimodal information during both the training and the testing processes [45]. A concatenation of specific modality attributes is the most basic example of a combined representation. The multimodal ‘m’ is described mathematically as $Y_{m}=f(Y_{1},Y_{2},\ldots ,Y_{n})$.

### 2.4. Data Acquisition

In this experiment, to create a multimodal system, we collected 3-channel EEG and audio recordings from the MODMA dataset [47]. EEG recordings include data from 55 participants; here, 26 depressed and 29 non-depressed participants were recorded. The data was recorded in a resting state (participants’ eyes were closed) for 90 s in a room with no noise [48]. A pervasive EEG collection device with three electrodes is used for data collection from the prefrontal lobe of participants because this part of the human brain relates to emotions and psychiatric conditions [49].

Audio recordings include data from 52 Chinese subjects (23 depressed and 29 healthy participants). These data were collected from the Second Affiliated Hospital of Lanzhou University [50]. In this dataset, each subject had 29 recordings, which were classified as positive, neutral, and negative emotional stimuli. High-quality equipment was used to collect speech data. All the participants from healthy and depressed groups were aged 18 to 55.

For data preprocessing, the Mel Frequency Cepstral Coefficients technique was implemented. Due to its efficiency in identifying the change of low-frequency data and focusing on people’s perceptions, the MFCC feature is the most commonly utilized audio feature in speech-related activities and signals like EEG waves. To avoid its large variance affecting the training, all MFCC factors were normalized. Thus, after preprocessing, 161 features were extracted using MFCC for both audio and EEG signals. Results are discussed in Table 4.

### 2.5. IIDs and Non-IIDs

**Figure 3:**
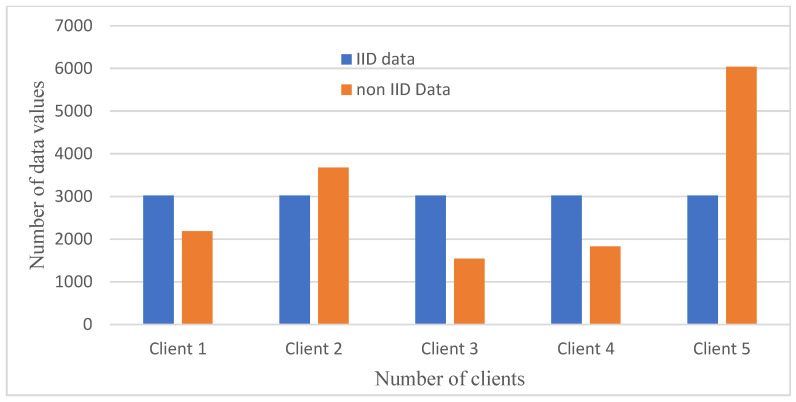
IID and non-IID data distribution of the EEG and audio dataset.

IID and non-IID data in FL represent various data distribution trends among the involved clients or systems. Every client or system in the federated learning configuration has an equivalent distribution of IID data available to it. The average distribution of the data is similar for all clients and is independent. However, non-IID denotes that data distribution among clients is neither identical nor independent. The data could be unbalanced, have distinct feature visualizations, or have multiple category distributions, among other statistical characteristics. The non-IID dataset was created by randomly distributing the original dataset among clients. Therefore, in non-IID, data is distributed in different patterns among clients. When a federated learning algorithm is combined with a deep learning algorithm, it effectively works on diverse datasets by involving relatively reliable clients as shown in Figure 3.

## 3. Results

In this section, the results of the DL algorithms LSTM, Bi-LSTM, and CNN are briefly discussed by comparing their performance in the classification of Major Depressive Disorder. By comparing the training and validation accuracy, the best-trained model was identified. Another FL-based deep multimodal model was trained for the classification of MDD. However, different Python libraries such as Pandas, Tensor-Flow, NumPy, Sklearn, and others were used for the preprocessing, categorization, scaling, testing, and training of the data.

### 3.1. Deep Learning for Audio Dataset

The DL algorithms are applied to the audio dataset after preprocessing the data to see the results in different situations with the best performance. In datasets related to depression, there is a significant data imbalance that brings a preference for non-depressed individuals in categorization. To reduce the prediction bias, sampling methods must be used to equalize the size of the depressed and non-depressed classes.

In addition, multiple signals from a single individual may emphasize very personal characteristics. Therefore, it is important to standardize the length of each person’s responses. MFCC is a commonly used voice descriptor for depression detection of depression. Thus, with the help of MFCC, 161 columns of features were extracted from the audio dataset. Subsequently, the DL models LSTM, CNN, and Bi-LSTM were used to classify depressed and non-depressed subjects.

**Figure 4:**
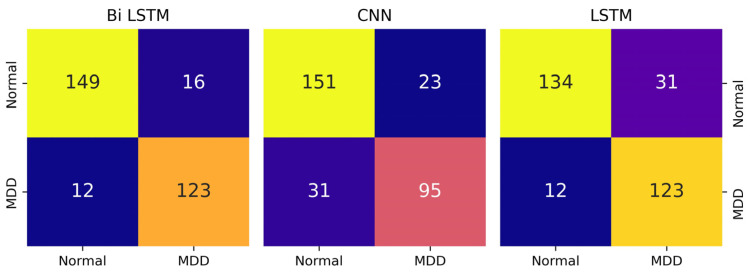
Confusion matrix of DL models with the audio dataset.

**Figure 5:**
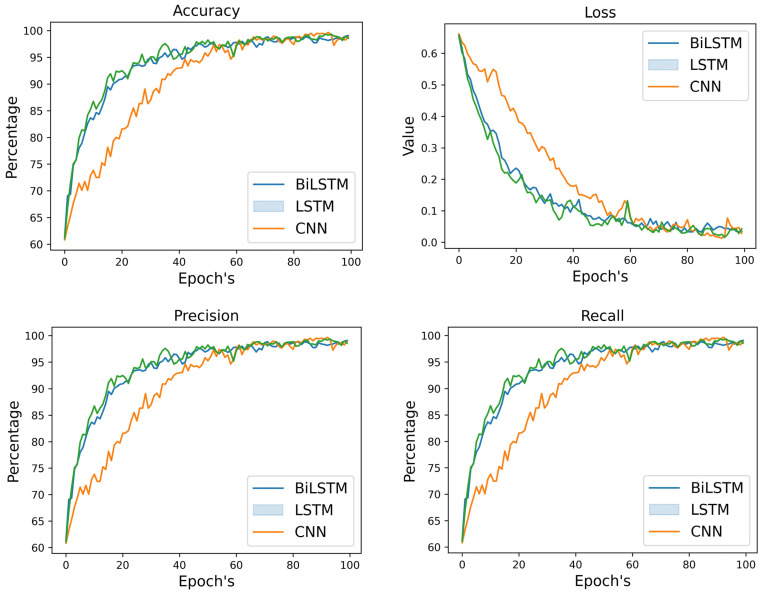

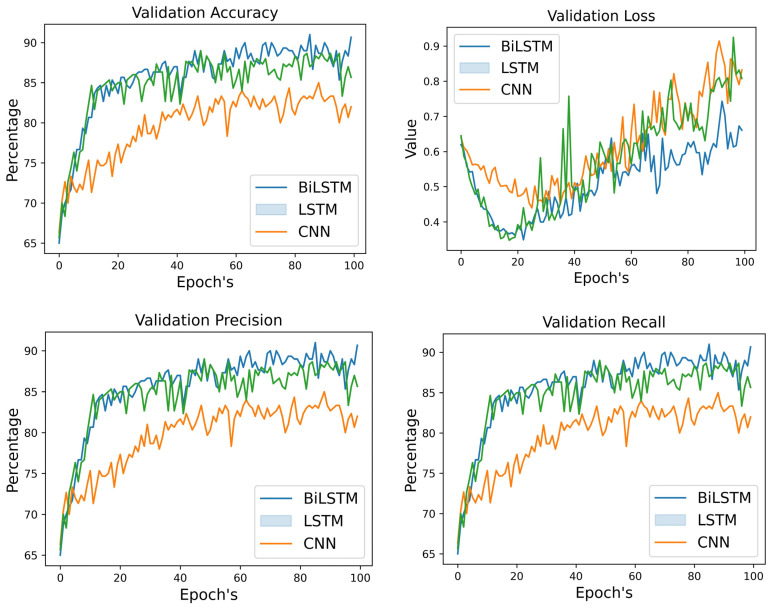
Training and Validation results using DL models for audio data.

Table 5 and Figure 5 displays the outcomes of comparing various DL algorithms working with audio features. Therefore, validation parameters clearly show that Bi-LSTM performed better upon audio features compared to the CNN and LSTM models. The Bi-LSTM model attained the highest validation accuracy, precision, and recall, with a score of 91% for each of these outcomes. On the other hand, LSTM and CNN models achieved validation accuracy of 89% and 85%, respectively. In Figure 4, the confusion matrix clearly shows that the Bi-LSTM model identified MDD and normal subjects more correctly than CNN and LSTM models using the audio dataset.

### 3.2. Deep Learning for EEG Dataset

EEG data is firstly preprocessed and 3-channel data is converted into 161 columns and 20,134 rows for the application of the DL model. EEG signals are one-dimensional data. For their classification, a one-dimensional model was developed using CNN, LSTM, and Bi-LSTM algorithms. However, the EEG data was collected from the subject’s prefrontal lobe using a 3-channel device that detects brain activities.

**Figure 6:**
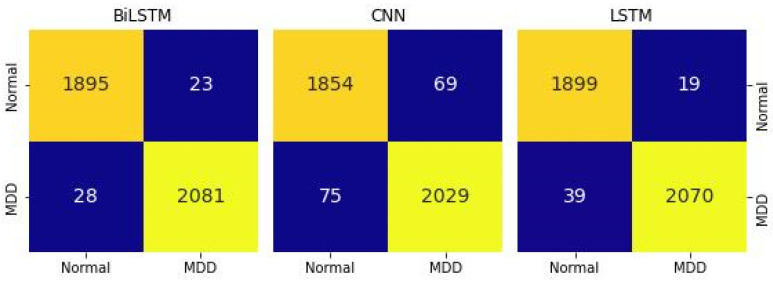
Confusion matrix of DL models with the EEG dataset.

The deep learning models gave better results with EEG data, as shown in Table 6. The training and validation results show that the Bi-LSTM and LSTM models performed approximately equally upon the EEG dataset. In Figure 6, confusion matrices present the number of identified MDD and normal patients with the help of different DL models using the EEG dataset.

**Figure 7:**
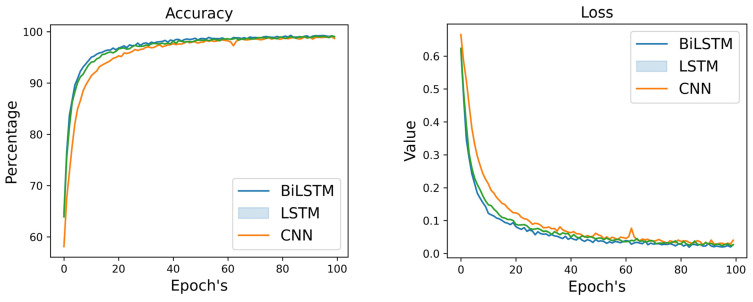

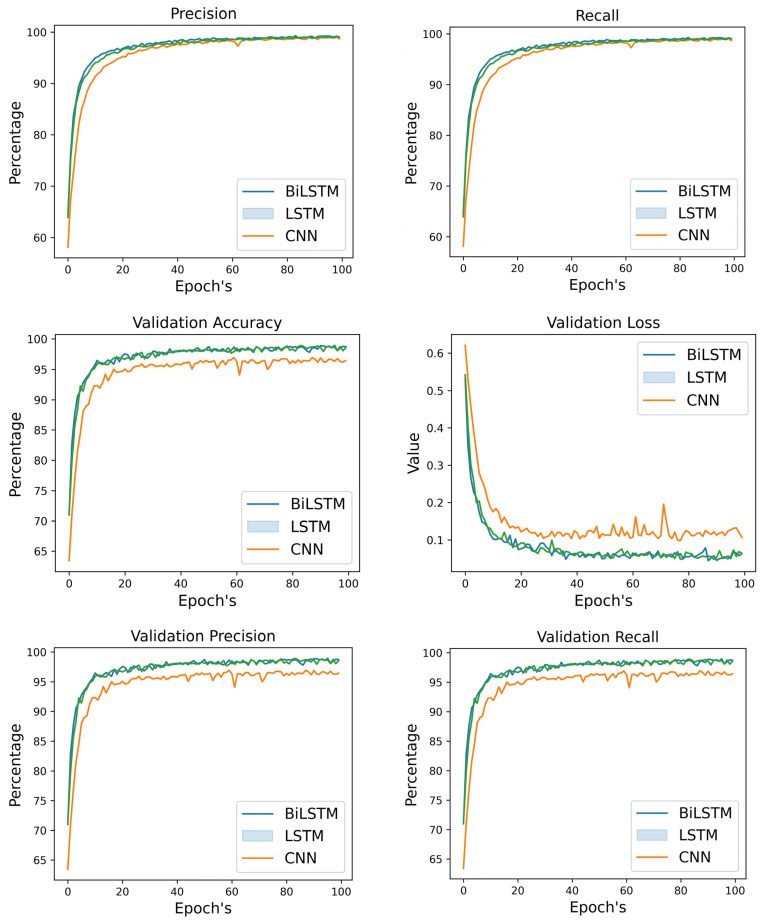
Training and Validation results using DL models for EEG data.

The results in Table 6 demonstrate that the validation accuracy, precision, and recall using the Bi-LSTM model is the highest with a score of 98.95% for each parameter. The LSTM model took second place with a score of 98.93% for each of the three parameters; the validation accuracy, precision, and recall. However, the CNN model has also achieved good validation results with 96.92% accuracy, precision, and recall. Figure 7, shows the results of the DL models increasing in value with every epoch from 0 to 100.

### 3.3. Deep Learning Using Multimodal Audio and EEG Datasets

Multimodal learning is the process of linking information from different sources. The fundamental challenge when using DL algorithms is to determine the best architecture for a target. A strong model that provides high accuracy in EEG identification may not perform as well on voice recognition tests. The aim of this work is to present a DL model for MDD classification to improve the accuracy of the classification problem. The DL algorithm Bi-LSTM was developed to train a multimodal model based on audio and EEG data.

For this purpose, the audio and EEG datasets are first preprocessed, and then the extracted features are collected in 161 rows for each dataset. To train a multimodal system, we need equal parameters. Thus, the concatenation layer merges both models in the form of a multimodal model.

**Figure 8:**
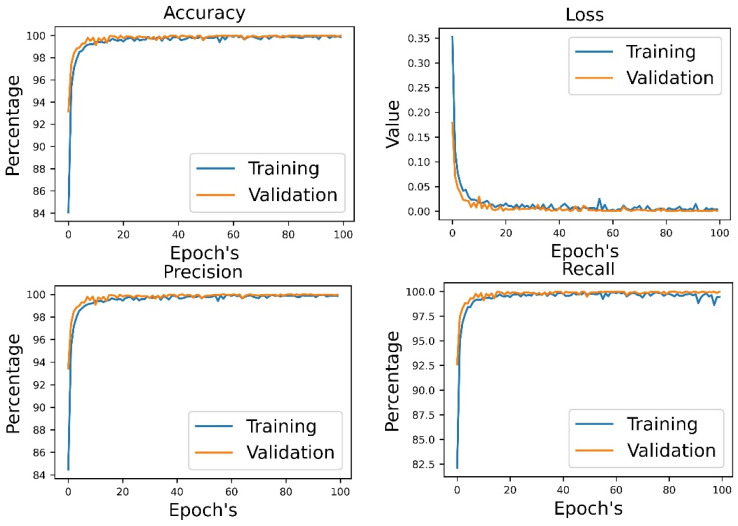
Training and validation results for multimodal DL using audio and EEG data.

Table 7 shows the results of the multimodal DL model with audio and EEG data. The validation results clearly show that it is a robust model with an accuracy, precision, and recall of 100% in each case. In Figure 8, we can see that the accuracy increases from the first epoch, and the differences between the training and validation accuracy are very small. This model could, therefore, be used in real-life situations.

### 3.4. Federated Learning Multimodal Using Audio and EEG Datasets

The FL model was used together with the DL model to increase the confidentiality of the data by developing our model at the client’s site. The weights of the central model are initialized randomly in the initial phase of the FL procedure. After initialization, the central server repeatedly contacts the clients at each communication cycle until the frame converges. There are mainly two FL settings for data partitioning: (1) the IID setting, in which the data is distributed independently and identically among the clients, and (2) the non-ID setting, in which each client has data from different distributions. The communication process, therefore, becomes slower as the size of the NN increases. However, in this framework, the communication parameters are not applied. Further, FL could be used for heterogeneous data as in non-IID environments. Non-IID data could affect the accuracy and precision of the model, but this largely depends on the type of data the clients have. If the clients have data from healthy control subjects, this would result in negative samples, whereas in contrast, the model would recognize a positive number of samples for depressed control subjects. This model performed better in both IID and non-IID situations, demonstrating the stability of the proposed MFL model.

**Figure 9:**
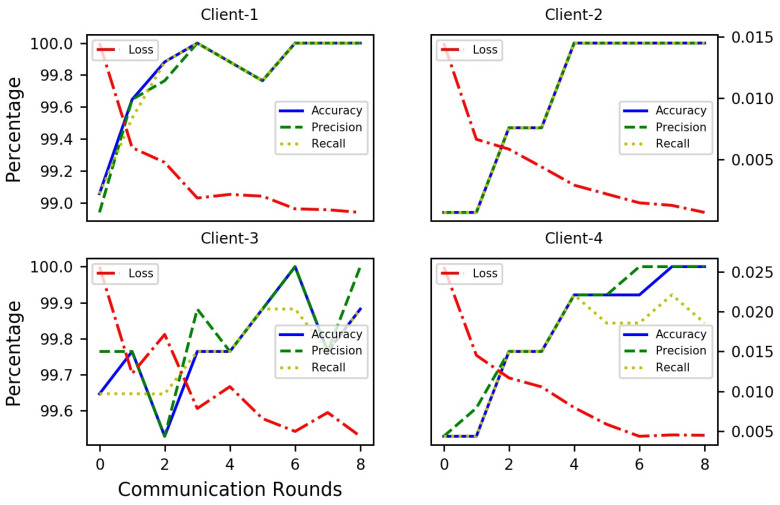

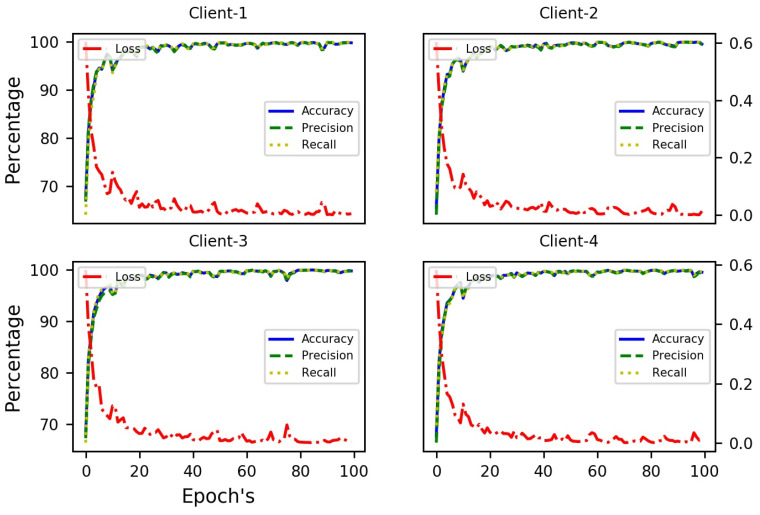
FL multimodal training and validation results using IID data.

As we can see from Figure 9 and Table 8, under the IID setting, the outcomes are slightly less than the central DL multimodal system because of the decentralized training of data at local locations. As such, the training and validation accuracies for all IID data are 99.94% and 99.93%, respectively. Further, the evaluation matrix’s result for validation precision is 99.9% and recall is 99.94%. Thus, a privacy-preserved FL model could be used for MDD diagnosis.

**Figure 10:**
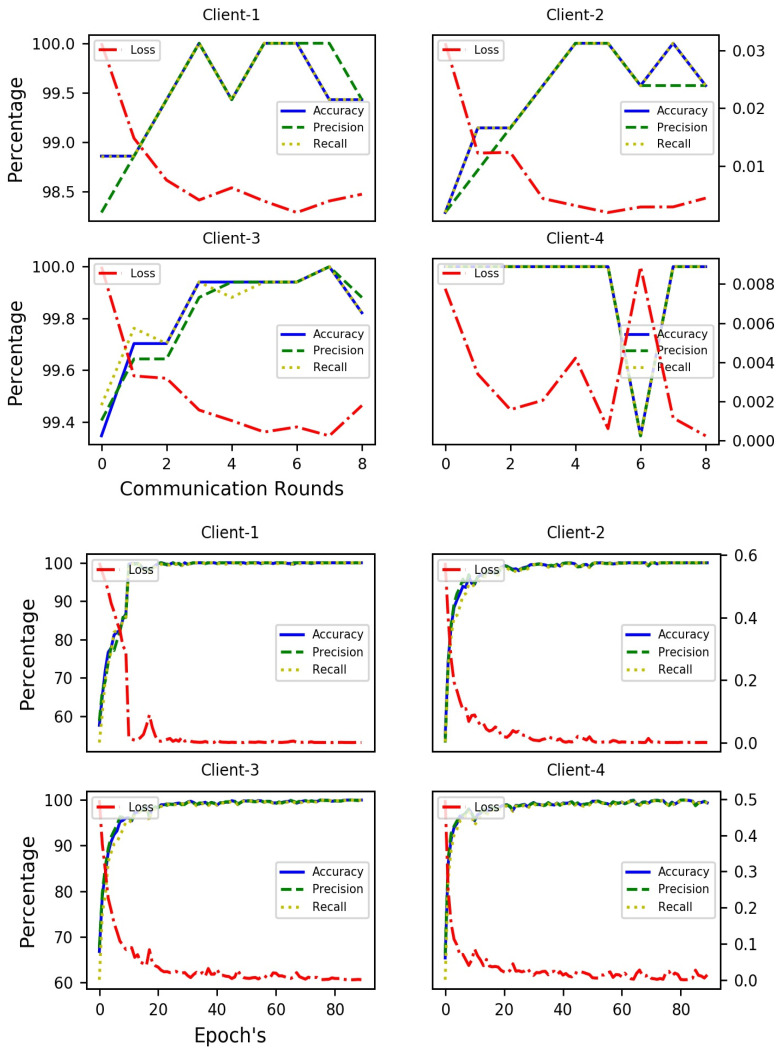
FL multimodal training and validation results using non-IID data.

Figure 10 and Table 9 show the performance of the FL multimodal system in non-IID settings. The results are slightly higher in non-IID settings than in IID settings. As such, the training accuracy, precision, and recall for non-IID settings are 99.99% for each. Further, we obtained 0 loss for training and validation.

## 4. Discussion

The performance of the proposed MFL model for detecting depression was compared with the pre-trained models in Table 1, Table 2 and Table 3. The proposed system performed better with more privacy by identifying depression with maximum accuracy and minimum loss using audio and EEG datasets. First, a base model with LSTM, Bi-LSTM, and CNN algorithms was trained individually on EEG and audio datasets. In both data sets, Bi-LSTM outperformed the other algorithms. Subsequently, a multimodal model was created from EEG and audio data sets by applying Bi-LSTM. The DL multi-model achieved 100% accuracy and precision in the validation phase. Finally, the FL algorithm was applied to the multimodal in combination with Bi-LSTM after splitting the EEG and audio datasets into four clients for IID and non-IID scenarios. The system captured the audio and EEG features more accurately with both IID and non-IID datasets. The proposed system achieved 99.9% accuracy with 0 losses in both IID and non-IIID scenarios. Thus, it is clear that the proposed system is consistent with training parameters with a wide set of clients.

## 5. Conclusions and Future Trends

In today’s world, it is crucial to classify MDD individuals while protecting privacy by using multimodal DL. In this study, the use of privacy-preserving federated deep learning for MDD classification was investigated. First, pre-trained deep learning models such as CNN, LSTM, and Bi-LSTM were used and evaluated. Compared to the other algorithms discussed, Bi-LSTM obtained a validation accuracy of 91% for audio data and 98.95% for EEG data. Consider the best performing model, i.e., Bi-LSTM, as the baseline model for multimodal deep learning. The multimodal DL model achieved a score of 100% validation accuracy, precision, and recall. In addition, a federated learning-based multimodal privacy-preserving model was implemented with Bi-LSTM for individuals with MDD using audio and EEG data. The proposed model could protect the high-risk and sensitive mental health data by developing it on the client’s side. Moreover, the concept of multimodality increases the robustness and reliability of the model by performing it with multiple data sources and types. For this experiment, we used the multimodal MODMA dataset. As a result, the proposed system achieved a training accuracy of 99.99% with 0 losses and a validation accuracy of 99.97% for IID and non-IID settings. Based on the results, it was found that the proposed system outperforms the federated multimodal deep learning system in terms of resource utilization and privacy while achieving classification results very similar to those of the baseline deep learning-based multimodal system. Most importantly, communication costs are lower in the FL model as the trained model is shared between client and server rather than the entire dataset. In future studies, this model could be applied to different types of datasets such as image data, video data, text data, etc.

## Figures and Tables

**Table 1:** Comparative analysis of previous studies’ results.

References	Data Type	Method Applied	Multi-Modality	Data Privacy	Parameters
[23]	Speech data	Deep Neural Network Architecture	No	No	96.7%
[24]	X, formerly Twitter data	Hybrid DL Model	No	No	F1 score 89%
[25]	EEG data	1D CNN Model	No	No	Accuracy 90.5%
[26]	EEG data	DL Models	No	No	Accuracy 99.24%
[27]	Text data	Deep Learning Model	No	No	Accuracy 99%
[28]	EEG data	Deep Belief Network (DBN) Model	No	No	Accuracy 83.16%, 86.09% under binary and multiple classes
[29]	X, formerly Twitter data	DL Models	No	No	Got high accuracy of 98% with CNN
[30]	Reddit data	EL Model	No	No	Accuracy 98.05%
[31]	Questionnaire data	AI-based Decision Support System	No	No	Accuracy 89%

**Table 2:** Depression detection using a Multimodal System.

References	Features Extracted	Method Applied	Data Privacy	Parameters
[33]	Social networks, visual, emotional, and user profile	A Multimodal Dictionary Learning	No	F1-measure is 85%
[34]	Audio, video, and text data	Multi-model Fusion	No	Root Mean Square Error is 5.98
[35]	Text, picture, and behaviour	Multimodal Feature Fusion Network	No	F1-score is 0.9685
[36]	Audio and text	Cross Dataset Prediction	No	Root Mean Square Error is 5.62 using Lenear Regression model
[37]	Audio and image	Time-Aware Attention-based Multimodal Fusion Depression Detection Network	No	F1-score is 0.75

**Table 3:** FL-based depression detection.

References	Data Type	Method Applied	Multi-Modality	Data Privacy	Parameters
[38]	Multi-source mobile health data	FL Model	No	Yes	Accuracy 85.13%
[39]	Speech data	FL Method	No	Yes	Accuracy 87%
[40]	Audio data	FL Model	No	Yes	Accuracy with IID 86.3% and with non-IID 85.3%
[41]	Text data	FL Framework	No	Yes	Accuracy 93.46%

**Table 4:** The details of participants from the audio and 3-channel EEG dataset.

Subject Type	Age (in Years)	Gender	
		Male	Female
Has Depression	16–56	15	11
No Depression	18–55	19	10

**Table 5:** Result table of DL algorithms for audio data.

Parameters	Bi LSTM	CNN	LSTM
Accuracy	99.08333	99.66667	99.33333
Val Accuracy	91	85	89
Precision	99.08333	99.66667	99.33333
Val Precision	91	85	89
Recall	99.08333	99.66667	99.33333
Val Recall	91	85	89
Loss	0.032376	0.012926	0.015737
Val Loss	0.349078	0.439554	0.347897

**Table 6:** Result table of DL algorithms for EEG data.

Parameters	BiLSTM	CNN	LSTM
Accuracy	99.28598	98.99417	99.19285
Val Accuracy	98.95704	96.92078	98.93221
Precision	99.28598	98.99417	99.19285
Val Precision	98.95704	96.92078	98.93221
Recall	99.28598	98.99417	99.19285
Val Recall	98.95704	96.92078	98.93221
Loss	0.019303	0.02646	0.023499
Val Loss	0.043929	0.09795	0.047406

**Table 7:** Result table of DL multimodal system.

Parameters	DL Multimodal
Accuracy	99.99411
Validation Accuracy	100
Precision	99.97054
Validation Precision	100
Recall	99.95877
Validation Recall	100
Loss	0.00047
Validation Loss	0.000117

**Table 8:** Result table of the FL multimodal system for IID settings.

IID Settings	
Parameters	FL Multimodal
Accuracy	99.94
Validation Accuracy	99.93
Precision	99.97
Validation Precision	99.9
Recall	99.88
Validation Recall	99.94
Loss	0
Validation Loss	0

**Table 9:** Result table of the FL multimodal system for non-IID settings.

Non-IID Settings	
Parameters	FL Multimodal
Accuracy	99.99
Validation Accuracy	99.97
Precision	99.99
Validation Precision	99.96
Recall	99.99
Validation Recall	99.95
Loss	0
Validation Loss	0

## Data Availability

The data presented in this study are openly available in https://modma.lzu.edu.cn/data/index/ (accessed on 18 December 2023) at [47].
